# Functional analysis of the Frzb2 gene in *Schistosoma japonicum*

**DOI:** 10.1186/s13567-019-0716-1

**Published:** 2019-12-11

**Authors:** Guifeng Cheng, Xiaochun Li, Fanglin Qin, Rong Xu, Yuanyuan Zhang, Jinming Liu, Shaopeng Gu, Yamei Jin

**Affiliations:** 1 College of Animal Science and Veterinary Medicine, Shanxi Agricultural University, Shanxi, China; 2 Key Laboratory of Animal Parasitology, Ministry of Agriculture of China, Shanghai Veterinary Research Institute, Chinese Academy of Agricultural Sciences, Shanghai, China; 3 College of Life Sciences, Shanghai Normal University, Shanghai, China

## Abstract

Schistosomiasis is a globally important helminthic disease of humans and animals, and it is the second most common parasitic disease after malaria. Eggs produced by mature females are responsible for the disease’s occurrence and spread. *Frzb2*, a secreted frizzled-related protein, can inhibit Wnt signalling by competitive binding to the specific frizzled protein receptor. In this study, the complete gene sequence of *SjFrzb2* was obtained by using 3′-rapid amplification of cDNA ends technology. *SjFrzb2* transcript levels at different stages of *S. japonicum* maturation were evaluated by quantitative real-time RT-PCR analysis. *SjFrzb2* was expressed at all developmental stages examined and exhibited the highest transcription level in 7-day-old worms, then gradually decreased during the growth and developmental stages to reach the lowest level at 18 days post-infection. *SjFrzb2* gene expression was higher in female worms than in male worms and was significantly higher in female worms from a single-sex infection than in female worms from a bisexual infection. The functions of *SjFrzb2* were explored via a small interfering RNA-based gene silencing approach and the soaking method. The results showed that *SjFrzb2* gene knockdown impaired the growth and development of *S. japonicum* in mice, affecting not only the survival and morphological structure of the worms but also their reproductive ability and the viability of the produced eggs. Collectively, these observations imply that Frzb2 may be a novel target for the development of immuno- and/or small molecule-based therapeutics to control schistosomiasis fecundity and transmission.

## Introduction

Schistosomiasis infects over 200 million people globally and is a major public health concern in more than 70 developing countries [1, 2]. Furthermore, schistosomiasis can contribute to carcinogenesis and to higher susceptibility to other pathogens such as human immunodeficiency virus [3, 4]. Schistosomiasis is also a concern for travellers and immigrants in nonendemic countries [5–7]. Three major species of schistosomes (*Schistosoma mansoni*, *S*. *haematobium*, and *S*. *japonicum*) infect humans, among which *S*. *japonicum* is the most prevalent in China, Indonesia, and the Philippines [8, 9]. Bisexually infected female worms paired with males can continuously release several thousand eggs each day for many months or even years [10]. The eggs produced by mature female worms are important for parasitic transmission and disease pathogenesis. Female worms from single-sex infections (SF) are in a continually immature state and are normally unable to lay eggs [11], thereby inhibiting the occurrence and spread of schistosomiasis.

The regulation of various signalling pathways is essential for the growth and development of organisms. Signalling pathways transmit a variety of molecules, such as extracellular hormones, growth factors, and cytokines, which trigger a series of reactions that participate in many important biological processes. Wnt signalling is a highly conserved pathway that plays pivotal roles in embryonic development as well as energy metabolism and balance [12, 13]. Previous studies have indicated that the Wnt signalling pathway is critical for the normal development of the mammalian reproductive system, especially the formation of Müllerian ducts, and participates in the regulation of follicle development, ovulation, and luteinisation in the establishment of normal pregnancy [14–16]. Studies have also indicated that the inhibition of Wnt protein expression affects the reproductive development of *S*. *japonicum*.

The members of the secreted frizzled-related protein (sFrp) family can inhibit Wnt signalling by competitive binding to the specific frizzled protein receptor (Fz receptor) [17]. sFrp family members exist in humans, chickens, mice, and frogs (genus *Xenopus*) [18, 19]. Frzb2 has been cloned in *Xenopus* and identified in the early anterior neural network of chickens. A previous study showed that ectopically expressed Frzb2 can inhibit head formation in *Xenopus* [20]. Moreover, a previous study by our group showed that the *Frzb2* gene is highly expressed in SF compared with mature females from a bisexual infection (MF) (data not published), suggesting that *SjFrzb2* is associated with the sexual maturity of female *S. japonicum* worms.

In the present study, the complete gene sequence of *SjFrzb2* was obtained by 3′-rapid amplification of cDNA ends (RACE) technology. *SjFrzb2* was analysed at the transcriptional level at different stages of *S. japonicum* maturity using small interfering RNA (siRNA) to explore the functional roles of *SjFrzb2* in growth, development, and fecundity.

## Materials and methods

### Ethics statement

All animal experiments were conducted in accordance with the guidelines of the Committee for the Care and Use of Laboratory Animals of the Shanghai Veterinary Research Institute, Chinese Academy of Agricultural Sciences (permit no. SYXK-2016-0010). The study protocol was approved by the Ethics and Animal Welfare Committee of the Shanghai Veterinary Research Institute, Chinese Academy of Agricultural Sciences (experiment no. SHVRI—mo—2018040809).

### Parasites and animals

The Chinese strain of *S. japonicum* used in this study was maintained at the National Institute of Parasitic Diseases, Chinese Center for Disease Control and Prevention. Freshly shed cercariae were obtained via the exposure of infected snails (*Oncomelania hupensis*) to light. Male BALB/c mice (*n* = 60; age, 4–6 weeks; Shanghai SLAC laboratory Animal Co., Ltd., Shanghai, China) were percutaneously infected with bisexual or single-sex cercariae of *S*. *japonicum*. The worms were collected by hepatic-portal perfusion at different times, as described elsewhere [21]. The worms were then washed three times with phosphate-buffered saline (PBS, pH 7.4) at 4 °C to remove residual host debris. The SFs were collected directly. Partial paired worms at 18, 21, 25, 28, 35, and 42 days post-infection (dpi) were carefully separated from male and female worms (MF).

### Complete coding sequence (CDS) analysis

The 3′-RACE primer GSP (5′-GAC TTC GAT GTT CCG GTT GTG TA-3′) was designed with Primer 5.0 software (Premier Biosoft, Palo Alto, CA, USA) based on a *SjFrzb2* reference sequence (GenBank accession no. AAX24900.2). Total RNA from 25 adult *S. japonicum* worms was extracted using TRIzol reagent (Invitrogen Corporation, Carlsbad, CA, USA) according to the manufacturer’s instructions. The 3′-ends of the cDNA molecules were synthesized using the First Choice^®^ RLM-RACE Kit (Invitrogen). The 3′ RACE-PCR products were purified and ligated into the pGEM T-easy vector (Takara Bio, Inc., Otsu, Shiga, Japan) and then transformed into competent *Escherichia coli* DH5α cells (Tiangen Biotech (Beijing) Co., Ltd., Beijing, China). Clones were selected and subjected to DNA sequence analysis. The complete sequences along with the reference sequence were submitted to the GenBank database (accession number: MK253101). The complete sequences were analysed using the ORF Finder (Open Reading Frame Finder) graphical analysis tool [22]. The molecular weight, isoelectric point, and amino acid composition were determined using the ProtParam computational tool [23]. Signal peptide prediction was performed with the SignalP 3.0 server [24]. Transmembrane helices were analysed using the TMHMM method for the prediction of transmembrane protein topology based on a hidden Markov model [25]. Conserved domains were identified using the Conserved Domain Database [26]. The amino acid sequences of three orthologues were retrieved from the GenBank database for further multiple alignment analysis against the homologous sequences of *Schistosoma mansoni*, *Mus musculus*, and *Homo sapiens* using DNAMAN software.

### Quantitative real-time PCR analysis (qRT-PCR)

Total RNA of *S. japonicum* at different stages was transcribed using the PrimeScriptTM^RT^ reagent Kit (Takara Bio, Inc.). The following primers were used for qRT-PCR analysis: *SjFrzb2*-F (5′-TGA GAC GTC CAC CTT CAC AA-3′)/*SjFrzb2*-R (5′-AGA CCG TCT AGT TGG TGT TG-3′), which amplified a 122-bp product of *SjFrzb2*; and *SjTub*-F (5′-ACC TCA ACA ACC ACC-3′)/*SjTub*-R (5′-TTG CGG CTT CTG CTC TTC-3′), which amplified a 234-bp product of the β-tubulin gene. qRT-PCR amplification was performed with the SYBR Premix Ex Taq™ kit (Takara Bio, Inc.) and an ABI7500 Fast Real-Time PCR System (Applied Biosystems, Carlsbad, CA, USA). All experiments were performed in triplicate, and relative gene expression levels were calculated using the tubulin gene as an internal standard.

### Selection of effective siRNA of SjFrzb2

Three specific siRNA pairs (*SjFrzb2*-S1, *SjFrzb2*-S2, and *SjFrzb2*- S3) were designed based on the *SjFrzb2* gene sequence (GenBank accession no. MK253101) (Table 1). Each siRNA targeted a different region of the *SjFrzb2* coding sequence. These siRNA pairs and a nonspecific (negative control, NC) siRNA pair (NC siRNA), with no homologous sequences in the schistosomal genome, were successfully used as control siRNA in other *S. japonicum* siRNA experiments [27–30] and were synthesized by Shanghai GenePharma Co., Ltd. (Shanghai, China). In this experiment, 10 mice were exposed to approximately 200 cercariae through shaved abdominal skin using the slide-cover-glass method and divided into five groups (2 mice/group). At day 22 dpi, the *SjFrzb2* siRNAs and NC siRNA (dissolved in 200 μL of diethyl pyrocarbonate-treated water) or PBS were rapidly injected via the tail vein. The final siRNA concentration adjusted to host blood was approximately 100 nM, and this concentration was confirmed to confer the greatest inhibitory effect against *S. japonicum* siRNA using the socking method [31]. After 48 h, parasites were recovered from the hepatic veins by perfusion. Then, total RNA was isolated and amplified by qRT-PCR to evaluate interference effects.

**Table 1:** **Sequences of** ***SjFrzb2*-specific siRNAs and the control siRNA**

Name	Sequence		Targeted regions*
	Sense	Anti-sense	
S1 siRNA	5′-GCGCACAGAUUGCAUAUAUTT-3′	5′-AUAUAUGCAAUCUGUGCGCTT-3′	95–112 bp
S2 siRNA	5′-GCUUCCAUCAACUAAUGUUTT-3′	5′-AACAUUAGUUGAUGGAAGCTT-3′	322–339 bp
S3 siRNA	5′-GGAGUAUUGCAUGUUGAAUTT-3′	5′-AUUCAACAUGCAAUACUCCTT-3′	441–458 bp
NC siRNA	5′-UUCUCCGAACGUGUCACGUTT-3′	5′-ACGUGACACGUUCGGAGAATT-3′	Not applicable

*Reference sequence MK253101.

### siRNA for the long-term knockdown of *SjFrzb2* gene expression

S1 siRNA was selected for long-term interference to evaluate the effects of *SjFrzb2* on the parasitic development of *S. japonicum*. Twelve mice infected with 40 ± 5 cercariae were divided into three groups (4 mice/group). Long-term dose administration was used to maintain the siRNA concentration. Starting at 4 dpi, each mouse received 10 injections of 1 OD *SjFrzb2*-S1, NC siRNA (dissolved in 200 μL of diethyl pyrocarbonate-treated water) or 200 μL PBS via the tail vein every 4 days, as described in a previous study [32]. At 42 dpi, parasites were recovered and counted. The livers were also collected from the infected mice. Silencing effects were evaluated by qRT-PCR, electron microscopy, and calculation of the liver egg-hatching rate. qRT-PCR was also performed to detect the transcriptional levels of Wnt signalling-related genes [i.e., *SjWnt1* (KM668879.1), *SjWnt2* (DQ643830), *SjWnt4* (DQ643829), and *SjWnt5* (KC707587.1)] in worms after treatment with *SjFrzb2*-S1 siRNA. The primer sequences for the Wnt-related genes are shown in Table 2.

**Table 2:** **Primer sequences for the amplification of** ***Wnt*** **signalling-related genes**

Name	Sequence		Amplicon length (bp)
	Sense	Anti-sense	
*Sj wnt1*	5′-ACAACGAAATCAACAACTTGCTCAC-3′	5′-AAGTCAGTGGATGGGAATGTAGAAG-3′	106
*Sj wnt2*	5′-AATCGTGTAACCAAATGTAAATGCC-3′	5′-CCAATCTTGGCTCATAAGTAACACG-3′	125
*Sj wnt4*	5′-TATCATCAGTAGTATCAGGAGTATC-3′	5′-TGGTGATGGTAAAGGCGATGTAGTC-3′	117
*Sj wnt5*	5′-ATAATAATAGAGCAGGTCGTTTGGC-3′	5′-CTTGACGAAGATAACGACCAATACG-3′	134

### Worm burden calculation, liver egg count, and miracidial hatching rate

The worm burden reduction was calculated by comparing the number of worms recovered from the specific siRNA group to that of the control group using the formula WBR = (1 − WRSG/WRCG) × 100%, where WRCG is the number of worms recovered from the control group, and WRSG is the number of worms recovered from the siRNA group.

To evaluate the liver egg burden, the liver of each mouse was removed, weighed, homogenized, and digested with 10% NaOH for 10 min at 56 °C. The eggs were counted, and the number of liver eggs per gram was compared to that of the control mice using the formula ERR = (1 − EPGSG/EPGCG) × 100%, where ERR is the egg reduction rate, EPGCG is the number of eggs/g in the control group, and EPGSG is the number of eggs/g in the specific siRNA group.

To hatch the miracidia, 4 mL of liver homogenate was added to a flask filled with chlorine-free water. The neck of the flask was filled with a very thin layer of absorbent cotton, and the flask was incubated at 25–30 °C in the light. The supernatant above the cotton, which included the miracidia, was collected at 4 h after hatching. The miracidia were fixed with iodine and collected by centrifuging the supernatant at 4000 × *g* for 5 min at 4 °C. The numbers of miracidia were counted under a light microscope to calculate the hatching rates (i.e., number of observed miracidia divided by the number of added eggs). The hatching rate reduction was calculated by comparison to the control group using the formula HRR = (1 − HRSG/HRCG) × 100%, where HRR is the hatching rate reduction, HRCG is the egg-hatching rate of the control group, and HRSG is the egg-hatching rate of the specific siRNA group.

### Scanning electron microscopy (SEM) and transmission electron microscopy (TEM)

Adult worms were collected from the infected mice by perfusion and cleaned with PBS (pH 7.4), then fixed in 2% paraformaldehyde and 2.5% glutaraldehyde at 4 °C for 24 h. For SEM, the samples were washed three times (15 min/time) with PBS, fixed in 1% osmium tetroxide for 2 h, and then dehydrated in a graded ethanol series of 30%, 50%, 70%, 80%, 90%, 95%, and 100% for 20 min each time. After desiccation in a CO_2_ critical point drying apparatus, the dehydrated worms were coated with a gold film in a vacuum-coating device and then examined under a JSM-6380LV scanning electron microscope (JEOL, Ltd., Tokyo, Japan). Micrographs were obtained at a magnification of 4000×. For TEM, the samples were washed with PBS, dehydrated in 50% ethanol, 70% ethanol, 90% ethanol, 45% ethanol/45% acetone, 90% acetone, and 100% acetone for 20 min each, and then soaked in pure acetone/embedding solution at a 3:1 dilution for 2 h, a 1:1 dilution for 4 h, and a 1:3 dilution overnight. The embedded specimens were cut into 60–100 nm-thick ultrathin sections using an ultramicrotome (EM UC7; Leica Microsystems, Wetzlar, Germany) and then double stained with uranyl acetate and lead citrate. Ultrastructural alterations were observed with a Tecnai G2 Spirit BioTWIN TEM system (FEI Electron Optics International B.V., Eindhoven, Netherlands).

### Statistical analysis

Data are presented as the mean ± standard deviation (SD). All statistical analyses were performed with Student’s *t* test or one-way analysis of variance. A probability (*p*) value of ≤ 0.05 was considered statistically significant.

## Results

### Complete CDS and bioinformatic sequence analysis of *SjFrzb2*

**Figure 1:**
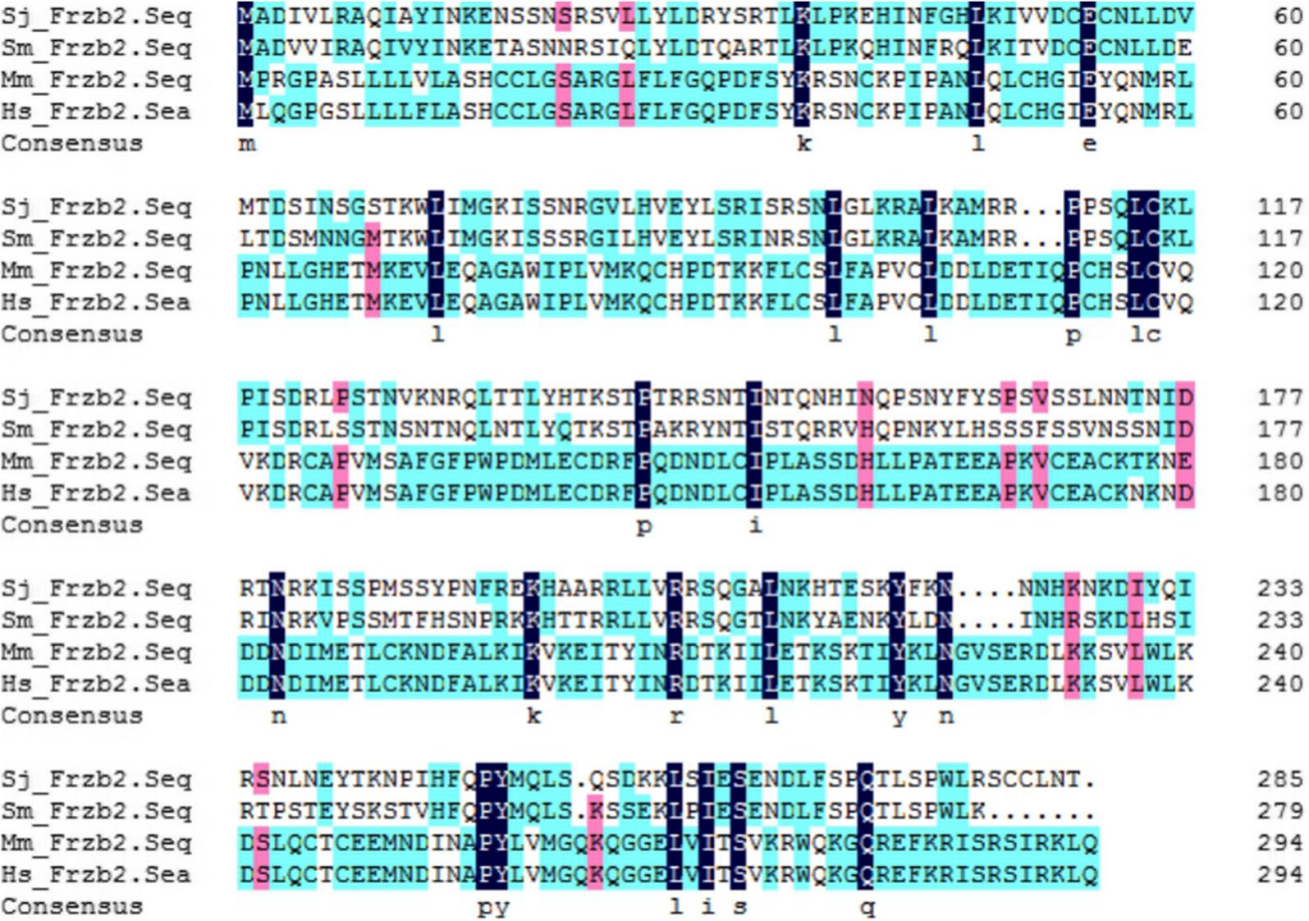
**The complete protein sequence of SjFrzb2 in relation to its orthologues in** ***S***. ***mansoni***, ***M***. ***musculus*, and** ***H. sapiens***. DNAMAN alignment of the derived amino acid sequences of SmFrzb2 (XP 018652209.1), MmFrzb2 (NP 033170.1), and HsFrzb2 (NP 003004.1). The regions with high identity and similarity between the Frzb2 sequences are shown in different colours (black, pink, and blue indicate similarity rates among amino acids at that position of 100%, 75%, and 50%, respectively).

The combination of the 3′ RACE-PCR product with the reference sequence (GenBank AAX24900.2) resulted in an *SjFrzb2* sequence with a total length of 1047 bp, which was submitted to the GenBank database under accession no. MK253101. The coding sequence of this gene consisted of 855 bp, encoding a protein comprising 284 amino acids with a molecular weight of approximately 36.5 kDa and an isoelectric point of 5.13. The sequence presented no signal peptide or transmembrane domain but did include a cysteine-rich Frizzled C-terminal domain and a conserved netrin module. A comparison of the amino acid sequences showed that the Frzb2 segment of *S. japonicum* shared 70%, 21%, and 21% identity with its orthologues in *S. mansoni*, *M. musculus*, and *H. sapiens*, respectively (Figure 1).

### *Frzb2* transcript levels in *S. japonicum*

The transcript levels of *SjFrzb2* were evaluated at different developmental time points, between the sexes, and at different maturation stages using qRT-PCR analysis. The following eight stages were examined: 7, 14, 18, 21, 25, 28, 35, and 42 days. After 18 days, the female and male worms were separated for examination. Additionally, the transcript levels of *SjFrzb2* at 25 days in SF and 25 days in MF were examined. Differences in transcript levels were observed by comparison with the housekeeping gene β-tubulin. All samples were run three times in triplicate, and Student’s *t*-test and one-way analysis of variance were used to analyse the data.

**Figure 2:**
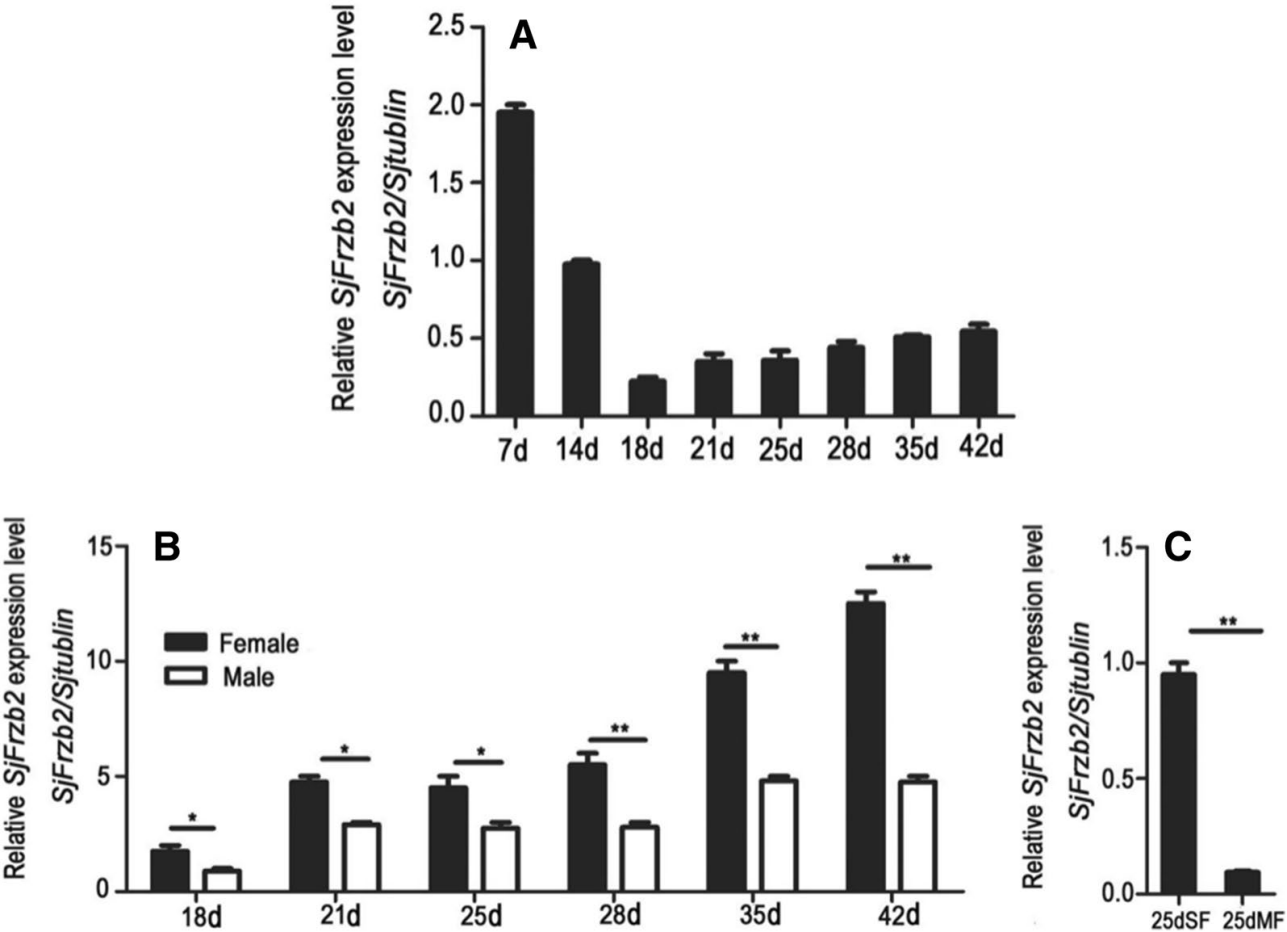
***SjFrzb2*** **mRNA profiles detected by qRT-PCR. A** A comparison of *SjFrzb2* transcript levels at 7, 14, 18, 21, 25, 28, 35, and 42 dpi. **B** *SjFrzb2* mRNA expression profiles in female worms and male worms at 18, 21, 25, 28, 35, and 42 dpi. **C** *SjFrzb2* mRNA expression profile at 25 days (d) in SF (25-day-old female worms from a single-sex infection) and 25 days in MF (25-day-old female worms from a bisexual infection). *S. japonicum* β-tubulin was used as an internal control. All experiments were performed in triplicate, *p < 0.05, **p < 0.01 vs. the control group.

The results demonstrated that the *SjFrzb2* mRNA was expressed in all developmental stages examined and exhibited the highest transcription level in 7-day-old worms. Overall, RNA expression gradually declined to the lowest level at 18 days and then somewhat increased (Figure 2A). The results also suggested that transcript levels were higher in female worms than in male worms (Figure 2B). *SjFrzb2* mRNA expression was significantly higher at 25 days in SF than at 25 days in MF (Figure 2C).

### Selection of an effective siRNA against *SjFrzb2*

**Figure 3:**
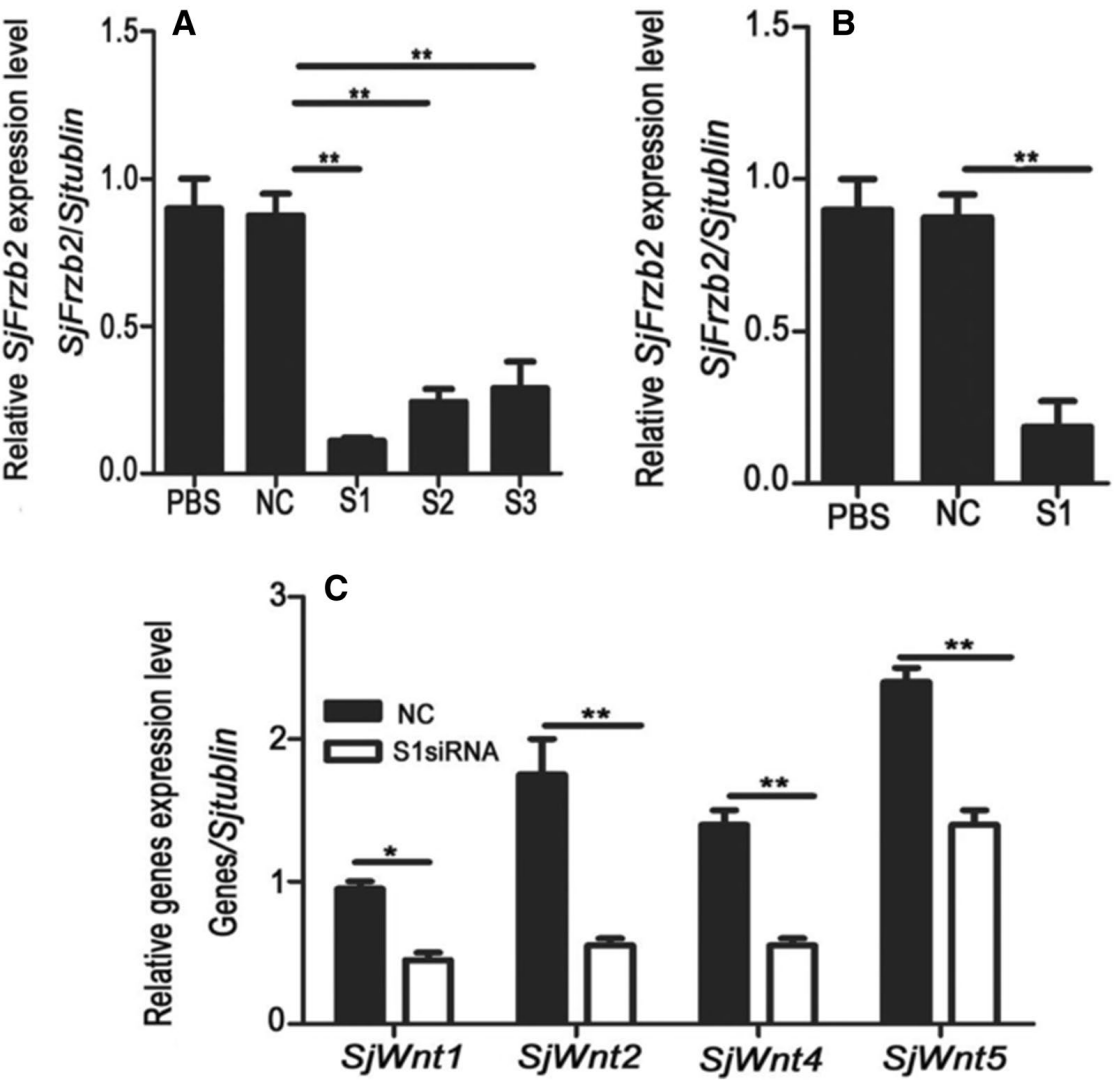
**Effects of siRNA on** ***SjFrzb2*** **expression normalized to** ***β*-*tubulin*** **mRNA. A** Comparison of the efficacies of the NC and three *SjFrzb2*-specific siRNA species (S1, S2, and S3) on *SjFrzb2* gene expression. **B** Effects of *SjFrzb2*-S1 siRNA on *SjFrzb2* gene expression in adult worms after the *SjFrzb2*-S1 treatment of mice at all developmental stages (10 injections at 4–42 dpi). **C** Expression levels of Wnt signalling-related genes in *SjFrzb2*-interfered worms. Data are presented as the mean ± SD of triplicate experiments. *p < 0.05, **p < 0.01 vs. the control group.

In the present study, the efficacy of three siRNAs (S1, S2, and S3) in the silencing of *SjFrzb2* were first tested in vivo. At 22 dpi, the siRNAs were injected into infected mouse hosts (~200 cercariae) via the tail vein, and the parasites were harvested after 48 h. The effects of gene silencing were measured by qRT-PCR. The median value of the NC siRNA group was set to 100%, and the inhibition efficiency in the siRNA-treated groups was calculated as described previously [33]. The abundance of the *SjFrzb2* gene transcript in the worms treated with the S1, S2, or S3 siRNA was reduced by 87.8%, 70.2%, or 71%, respectively, relative to the NC siRNA group (Figure 3A). Hence, the S1 siRNA was adopted to silence the expression of the *SjFrzb2* transcript in the following long-term siRNA experiment.

### *SjFrzb2* knockdown affects *S*. *japonicum* vitality, development, and reproduction

For the long-term (42-day) siRNA experiment, infected mice were injected 10 times with *SjFrzb2* S1 siRNA from 4 to 42 dpi every 4 days. Long-term treatment reduced *SjFrzb2* transcript levels by 73% compared with the NC siRNA group (Figure 3B). *SjFrzb2* silencing negatively affected parasitic vitality, development, and reproduction in mice. Compared with the NC and PBS groups, 45.95% and 35.49% of *S. japonicum* treated with *SjFrzb2* in hosts did not survive to adulthood (Table 3). *SjFrzb2* knockdown caused a decreases in the reproductive capacity of the surviving adult female worms of 33.28% and 46.70% compared with the NC and PBS groups, respectively. The loss of worms and reproductive capacity led to a lower liver egg burden. The liver egg burden reductions in the S1 siRNA group were 64.29% and 48.30%, while 62.24% and 61.71% of liver eggs failed to hatch into miracidia compared with the NC and PBS groups, respectively.

**Table 3:** **The effect of long-term** ***SjFrzb2*** **interference on the vitality and fertility of** ***S. japonicum***

Group (*n* = 4)	Number of adults (x ± s)	Average egg burden/female worm (x ± s)	Average egg burden/gram liver (x ± s)	Hatchability (x ± s)
PBS	31 ± 8.718	1991 ± 1014	18,983 ± 2250	0.1954 ± 0.0322
NC	37 ± 2.646	1591 ± 135	27,484 ± 784	0.1981 ± 0.0074
S1	20 ± 2.449	1061 ± 190	9814 ± 2202	0.0748 ± 0.0098
% reduction	35.49%** (PBS) 45.95%** (NC)	46.70%** (PBS) 33.28%** (NC)	48.30%** (PBS) 64.29%** (NC)	61.71%** (PBS) 62.24%** (NC)

** *p* < 0.01.

### *SjFrzb2* silencing leads to morphological changes in *S. japonicum*

**Figure 4:**
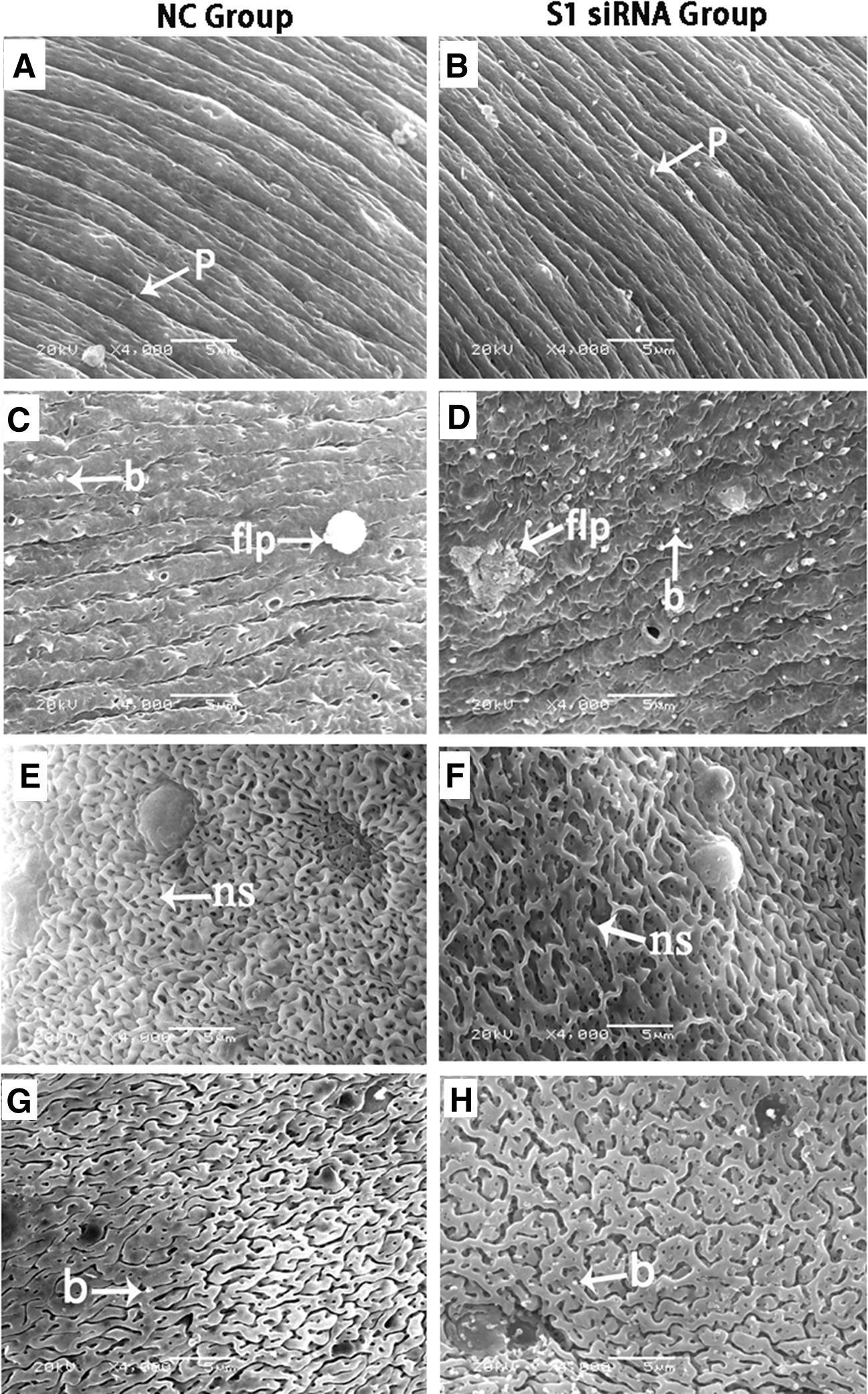
**Comparison of surface structures by SEM between 42-day-old adult worms in the NC group vs. the S1 siRNA group (10 injections via the tail vein at 4–42 dpi).** Compared with the control siRNA group, *SjFrzb2*-S1 siRNA induced wall disarrangement of females with notably larger wall protrusions (**A**, **B**); increased bubbles, and flower-like papillae with abnormal structures between the oral and ventral suckers of female worms (**C**, **D**). The surface appearance was similar, with disordering of the network structure of the gynaecophoral canal posterior portion (**E**, **F**). The bubbles were obviously larger on the distal-end body wall of males (**G**, **H**). flp: flower-like papillae, ns: network structure, p: protrusion, b: bubbles.

**Figure 5:**
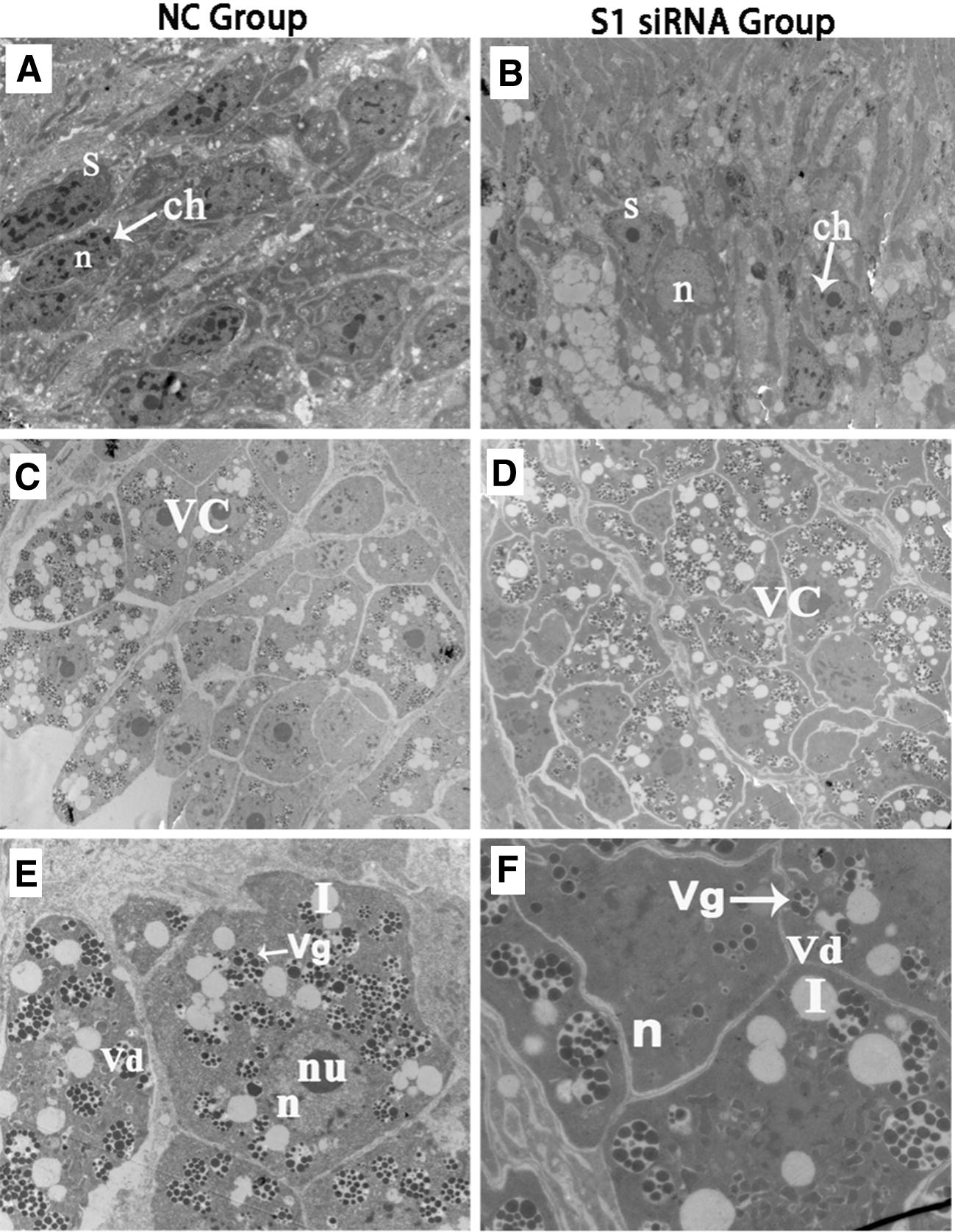
**Comparison of reproductive glands by TEM between 42-day-old adult worms in the NC group vs. S1 siRNA group (10 injections via the tail vein at 4–42 dpi).** *SjFrzb2*-S1 siRNA-induced alterations of the reproductive system are obvious. **A** Normally developed males exhibited greater numbers of spermatocytes, with chromatin around the nuclei. **B** The spermatocyte number was decreased, and the abundance of intracellular chromatin was reduced in the siRNA-treated groups. **C**, **E** Normally developed females presented mature vitelline cells, with regular cell edges and large numbers of vitelline droplets and globules. **D**, **F** Females in the siRNA-treated groups presented developmentally immature vitelline cells with fewer vitelline droplets and globules. vc: vitelline cells, vg: vitelline globules, vd: vitelline droplets, l: lipid droplets, n: nucleus, nu: nucleolus, s: spermatoblast.

*SjFrzb2* silencing also caused significant deformations of the adult worms. As shown in Figure 4, in comparison with the control siRNA group, the wall arrangement of females following *SjFrzb2* siRNA treatment was disordered, and the wall protrusions were notably larger (Figures 4A and B). The bubbles were obviously increased, and the flower-like papillae exhibited abnormal structures (Figures 4C and D). The appearance of the surfaces of the siRNA treated male worms were similar, with disordering of the network structure (Figures 4E and F) and obviously larger bubbles (Figures 4G and H). In regard to the reproductive system, normally developed males presented greater numbers of spermatocytes, with chromatin around the nuclei, while the spermatocyte number was decreased, and the amount of intracellular chromatin was reduced in the siRNA-treated groups (Figures 5A and B). The normally developed females exhibited mature vitelline cells with regular shapes of the cell edges and large numbers of vitelline droplets and globules, while females in the siRNA-treated groups exhibited developmentally immature vitelline cells with fewer vitelline droplets and Figures 5C–F.

### Effects of *SjFrzb2* silencing on the transcript levels of Wnt signalling-related genes

Because *Frzb2* can inhibit Wnt signalling by competitive binding to the specific Fz receptor, a long-term interference experiment was conducted to detect the effect of *SjFrzb2* silencing on Wnt signalling-related genes. Compared with the NC group, the expression levels of *SjWnt1*, *SjWnt2*, *SjWnt4*, and *SjWnt5* were decreased by 50.24%, 70.16%, 59.88%, and 42.65%, respectively, as a result of *SjFrzb2* gene interference (Figure 3C).

## Discussion

The large number of eggs produced by mature female worms of *S. japonicum* is primarily responsible for the disease pathology and is critical for the dissemination of schistosomiasis [10]. During growth and development, the female worm undergoes very significant physiological, morphological, and immunoreactivity changes, including body wall transformations, cell differentiation, intestinal junction formation, and organogenesis [11]. Wnt signalling is critical during this process. The Frzb2 protein, as a part of the fine regulatory mechanism of Wnt signalling, plays a very important role in signal transmission [17]. The high expression of the SjFrzb2 protein in early stages, female worms, and SF worms (Figure 2) fully demonstrated its importance in the growth and development of *S. japonicum*, especially in females. Moreover, *SjFrzb2* gene expression affects the expression patterns of other Wnt-related genes.

In schistosomes, the tegument is critical for nutritional uptake and signal transduction [34]. Since sexual maturation and spawning requires continuous male and female pairing, this interaction is necessary for the complete maturation of the vitelline gland and ovary and to ensure the normal development of egg cells and vitelline cells. Abnormal tegument structures may affect information transmission between males and females and inhibit the normal absorption of glucose and amino acids. In the present study, *SjFrzb2* knockdown not only caused significant morphological changes in the tegumental structure and gonadal cells of the parasites subjected to RNA interference (Figures 4, 5) but also reduced the survival and fecundity of the worms, which, in turn, reduced the host liver egg burden and egg viability (Table 3). Collectively, these effects contributed to the inhibition of the host pathological response and, subsequently, disease transmission.

In schistosomiasis, a vaccine that induces even a partial reduction in the worm burden could considerably reduce the pathology and limit parasitic transmission. Many potential vaccine antigens have been evaluated, but none have demonstrated satisfactory protection when used in the clinic [35]. The level of protection obtained by vaccination with these antigens rarely exceeded the 40% benchmark set by the World Health Organization. At present, there is no commercial vaccine available, even though several candidate antigens have been suggested by the World Health Organization or reported by researchers. The best long-term strategy for controlling schistosomiasis is through immunization with an anti-schistosomiasis vaccine combined with drug treatment. *SjFrzb2* is important for the growth, development, and reproduction of *S. japonicum*, as indicated not only by its mRNA expression profile, which is high in early schistosomules that are physiologically active, but also by the long-term siRNA experiment, in which the parasites subjected to RNA interference exhibited reductions in survival, fecundity, the live egg load, and egg-hatching rates. Based on these results, we propose that *SjFrzb2* should be further explored as a novel target for the development of immuno- and/or small molecule-based therapeutics to control schistosomiasis fecundity and transmission.
